# Promising derivatives of rutaecarpine with diverse pharmacological activities

**DOI:** 10.3389/fchem.2023.1199799

**Published:** 2023-11-01

**Authors:** Deping Li, Ziqian Huang, Xiaojun Xu, Yan Li

**Affiliations:** ^1^ Department of Pharmacy, First Affiliated Hospital of Gannan Medical University, Ganzhou, China; ^2^ Department of Party and Government Office, First Affiliated Hospital of Gannan Medical University, Ganzhou, China

**Keywords:** rutaecarpine, alkaloid, natural products, bioactivity, evodiamine, β-carboline

## Abstract

Rutaecarpine (RUT) is a natural pentacyclic indolopyridoquinazolinone alkaloid first isolated from one of the most famous traditional Chinese herbs, *Evodia rutaecarpa*, which is used for treating a variety of ailments, including headaches, gastrointestinal disorders, *postpartum* hemorrhage, amenorrhea, difficult menstruation, and other diseases. Accumulating pharmacological studies showed that RUT possesses a wide range of pharmacological effects through different mechanisms. However, its poor physicochemical properties and moderate biological activities have hampered its clinical application. In this regard, the modification of RUT aimed at seeking its derivatives with better physicochemical properties and more potency has been extensively studied. These derivatives exhibit diverse pharmacological activities, including anti-inflammatory, anti-atherogenic, anti-Alzheimer’s disease, antitumor, and antifungal activities via a variety of mechanisms, such as inhibiting cyclooxygenase-2 (COX-2), acetylcholine (AChE), phosphodiesterase 4B (PDE4B), phosphodiesterase 5 (PDE5), or topoisomerases (Topos). From this perspective, this paper provides a comprehensive description of RUT derivatives by focusing on their diverse biological activities. This review aims to give an insight into the biological activities of RUT derivatives and encourage further exploration of RUT.

## 1 Introduction

**FIGURE 1:**
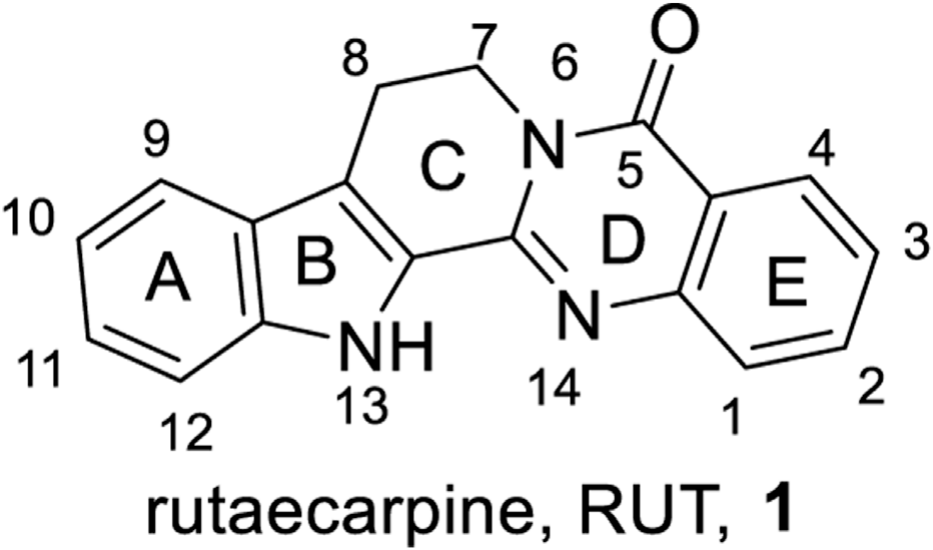
Structure, numbering atoms, and rings of RUT (**1**).

Rutaecarpine [8,13-dihydroindolo (2',3': 3,4) pyrido (2,1-*b*) quinazolin-5 (7*H*)-one, RUT, 1, Figure 1] is a pentacyclic indolopyridoquinazolinone first isolated by Asahina and Kashiwaki from *Evodia rutaecarpa* (Qiu, 2012), which is one of the most frequently used traditional Chinese herbs for treating diverse ailments, including headaches, gastrointestinal disorders, *postpartum* hemorrhage, amenorrhea, difficult menstruation, and other diseases (Lee et al., 2008; Liao et al., 2011). As one of the most abundant compounds in *Evodia rutaecarpa*, RUT has also been demonstrated to possess a broad spectrum of intriguing pharmacological activities, including anti-inflammation (Moon et al., 1999), anti-platelet (Sheen et al., 1996; Sheu et al., 1996), vasodilatory (Chiou et al., 1994; Li et al., 2021), analgesic (Zhang et al., 2017; Zhang et al., 2020), cytotoxic (Byun et al., 2022; Chan et al., 2022), anti-AD (Zhao et al., 2021), and anti-obesity activities (Kim et al., 2009), as well as anti-diabetic potential (Surbala et al., 2020; Xie et al., 2020). Nevertheless, RUT is still unsuitable for direct clinical application due to its poor water solubility, moderate potency, or cytotoxicity (Lee et al., 2017; Luo et al., 2020). From this perspective, the modification of RUT aimed at seeking its derivatives with better physicochemical properties (water solubility, oral bioavailability, etc.), more potency, and less cytotoxicity to normal tissues has been extensively explored.

In the past few years, there have been several reviews on RUT. In 2008, Lee *et al.* provided a review on the isolation, synthesis, structure–activity relationship (SAR) studies, pharmacological activities, and metabolism of RUT (Lee et al., 2008). Two years later, Jia et al. summarized its pharmacological effects as a cardiovascular protective agent (Jia and Hu, 2010). In 2015, Son *et al.* gave an updated review of RUT on its synthesis, biological activities, and SAR of RUT derivatives (Son et al., 2015). In 2019, Tian et al. gave an updated review of RUT as a promising cardiovascular protective agent (Tian et al., 2019). Recently, Li et al. comprehensively summarized the recent progress of RUT as a promising hepatoprotective agent (Li et al., 2020). These aforementioned reviews were either limited to RUT itself (its derivatives were not described) or to one type of pharmacological activity (such as cardiovascular protective activity or hepatoprotective activity), and recent studies were not included.

**FIGURE 2:**
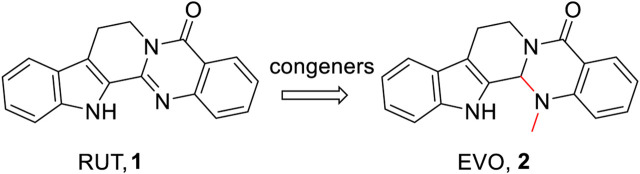
Congeners of RUT and EVO isolated from *Evodia rutaecarpa*.

Previously, a comprehensive description of the derivatives of evodamine (EVO, **2**, a congener of RUT, which is also one of the most bioactive alkaloids isolated from *Evodia rutaecarpa*) was conducted by our group (Li et al. 2022). In this study, we focus on the broad spectrum of intriguing biological activities of RUT derivatives, aiming to provide a reference for further studies of RUT (Figure 2).

## 2 Bioactivities of RUT derivatives

### 2.1 Anti-inflammatory activity

#### 2.1.1 Protecting against alcohol-induced liver injury

Alcoholic liver disease (ALD) represents the main type of chronic liver disease in the world (Seitz et al., 2018). It has been reported that cyclooxygenase-2 (COX-2) plays a vital role in the pathogenesis of ALD (Dunn and Shah, 2016). Enhanced expression of COX-2 can be seen in ALD (Nanji et al., 1997), and downregulating COX-2 can reduce inflammation and oxidative stress (Deol et al., 2018). Hence, an inhibitor of COX-2 may be an appropriate choice for the treatment of ALD.

**FIGURE 3:**
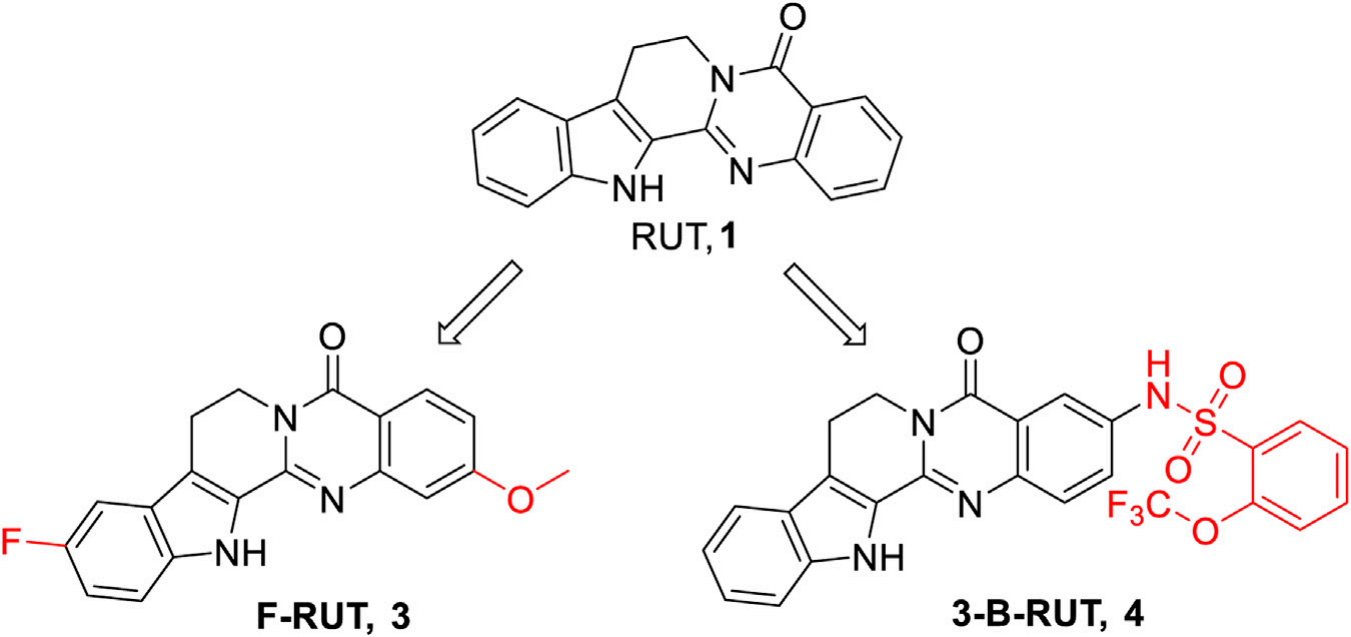
Structure of the representative compounds **3** and **4** with anti-inflammatory activity.

Previously, Lee and coworkers demonstrated that 10-fluoro-2-methoxyrutaecarpine (**3**) (Figure 3) retained anti-inflammatory activity both *in vitro* and *in vivo* (Lee et al., 2019). Based on the results, they further found that **3** exhibited better COX-2 suppression activity and lower cytotoxicity compared to its parent RUT as a COX-2 selective inhibitor.

Another team, Xu and coworkers, introduced a sulfonyl group to RUT to improve the anti-inflammatory effect and obtained a novel analog termed **3-B-RUT** (**4**) (Figure 3) (Xu et al., 2021). Then, they explored its protective effect on alcoholic liver injury (ALI) *in vivo* and *in vitro*. The results showed that **4** (20 μg/kg) attenuated ALI and suppressed oxidative stress and liver inflammation, and the effect was comparable to that of RUT (20 mg/kg). Mechanistically, the results of Western blot showed that administration of **4** inhibited the phosphorylation of P65 and IκBα (silencing COX2 also achieves this effect), leading to suppressing the transfer of P65 into the nucleus. As is known, NF-κB commonly refers to the dimer protein formed by the P65/P50 subunits, and it controls DNA transcription, cytokine production, cell survival, and other important cell events; it specifically plays a key role in regulating immune development, immune responses, oxidative stress, and inflammation (Mitchell et al., 2016). Therefore, **4** might suppress oxidative stress and inflammation by regulating the COX-2/NF-κB pathway. The increased anti-inflammatory activity leading to a lower concentration may overcome the undesirable side effects, such as in gastrointestinal or heart diseases. Therefore, **4** was considered to be a promising clinical candidate for the treatment of ALI.

#### 2.1.2 Alleviating acute kidney injury

Acute kidney injury (AKI) is a clinical syndrome caused by multiple factors, including ischemic–reperfusion injury, sepsis, and drug toxicity (such as cisplatin). Similarly, inflammation and oxidative stress have been shown to play critical roles in the pathogenesis of AKI (Liu et al., 2020).

**FIGURE 4:**
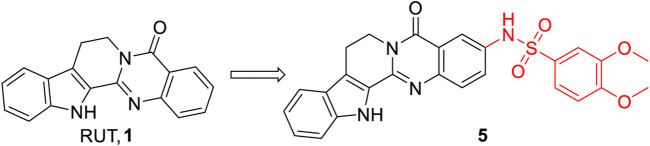
Structure of the representative compound **5** with anti-inflammatory activity.

Liu et al. found that phosphodiesterase 4B (PDE4B), a key enzyme modulator in immune and inflammation-related diseases, was significantly upregulated in the serum of AKI patients and participated in the progression of AKI (Liu et al., 2020). Therefore, they synthesized and obtained a series of 3-aromatic sulphonamide-substituted RUT derivatives, and compound **5** (Figure 4) exhibited the best renoprotective and anti-inflammatory activity (Liu et al., 2020). Using target prediction (performed by Discovery Studio 2017 software), molecular docking (pdb code: 5O0J), and cellular thermal shift assay (CETSA), they identified PDE4B as the target of **5**. Therefore, **5** might serve as a therapeutic candidate for AKI via the PDE4B pathway.

#### 2.1.3 Anti-atherogenic activity

Atherosclerosis is a chronic disease featuring lipid deposition and an inflammatory response (Libby et al., 2011). It is well known that nitric oxide (NO) and tumor necrosis factor (TNF)-α play vital roles in inflammation (Kaltenmeier et al., 2022). Furthermore, the NLRP3 inflammasome, a protein complex associated with the inflammatory progression of atherosclerosis, has attracted much interest in the pathogenesis of atherosclerosis (Abderrazak et al., 2015; Guo et al., 2015; Grebe et al., 2018). Therefore, compounds with reduced effects on NO, TNF-α, or NLRP3 that alleviate vascular inflammation would be beneficial for anti-atherogenic progression.

**FIGURE 5:**
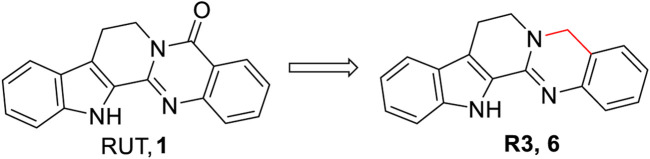
Structure of the representative compound **6** with anti-inflammatory activity.

Previously, Lee et al. (2017) reported that compound **3** could also inhibit the production of NO and TNF-α in lipopolysaccharide (LPS)-stimulated RAW264.7 macrophages. In addition, no cytotoxicity was observed toward RAW264.7 macrophages at a concentration of 20 µM. Although Luo et al. first demonstrated that 5-deoxy-rutaecarpine (R3, **6**) (Figure 5), a novel derivative of RUT, could alleviate atherosclerotic burden in Apoe^−/−^ mice fed a high-fat diet, pharmacokinetics studies showed that **6** had better oral bioavailability (*F*% = 27.89) than RUT (*F*% = 0.07) in male rats via gastric gavage at a dosage of 20 mg/kg. Subsequently, they explored its anti-atherosclerotic mechanisms (Luo et al., 2020). The results showed that **6** inhibited NLRP3 inflammasome activation through NF-κB, and the MAPK signaling pathway blocked atherosclerotic progression via debilitating NLRP3 inflammasome-related inflammation.

**FIGURE 6:**
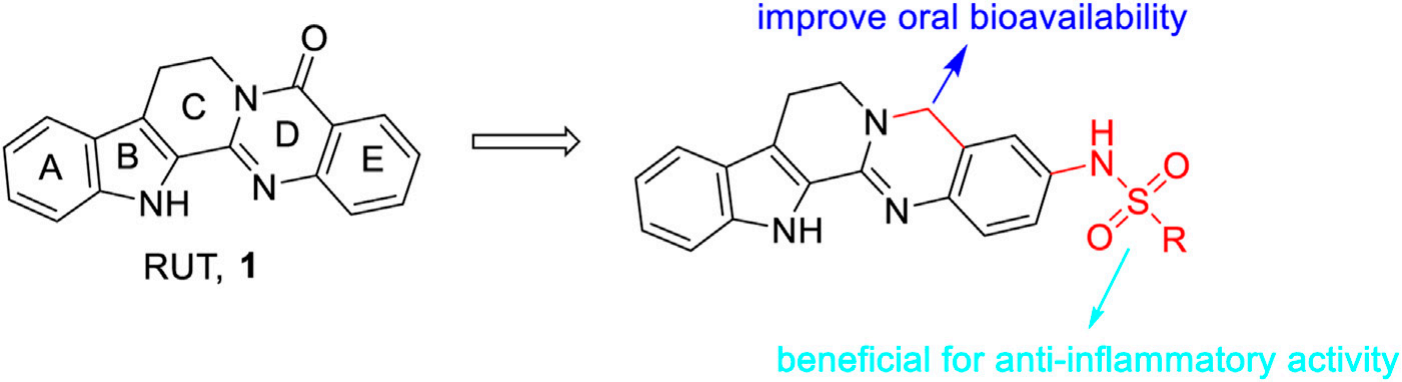
General SAR observations for anti-inflammatory activity.

Based on the aforementioned information, we found that i) modification of the D-ring of RUT, especially the introduction of a sulfonyl group to the D-ring of RUT, seemed beneficial to enhance its anti-inflammatory activity; and ii) removal of the carbonyl group from the C-ring of RUT may improve its oral bioavailability (Figure 6).

### 2.2 Anti-Alzheimer’s disease activity

Alzheimer’s disease (AD) is an incurable and long-term neurodegenerative brain disorder, and its pathogenic mechanism is still not fully understood. Currently, many hypotheses, including low levels of acetylcholine (AChE) and butyrylcholinesterase (BChE), aggregation of tau protein, and deposition of amyloid β-protein (Aβ) plaques, etc., have been proposed to explain the pathophysiology of AD (Wilson et al., 2012). Currently, the development of anti-AD drugs mainly focuses on reducing the levels of AChE and/or BChE, Aβ, and tau proteins.

#### 2.2.1 As AChE inhibitors

Although **6** was demonstrated to exhibit anti-atherogenic activity, it is worth noting that it was first synthesized by Decker in early 2005, and its cholinesterase inhibitory activity was evaluated (Decker, 2005). The results showed that **6** exhibited moderate inhibitory activity toward both AChE and BChE with IC_50_ values of 3.4 μM and 0.5 μM, respectively.

**FIGURE 7:**
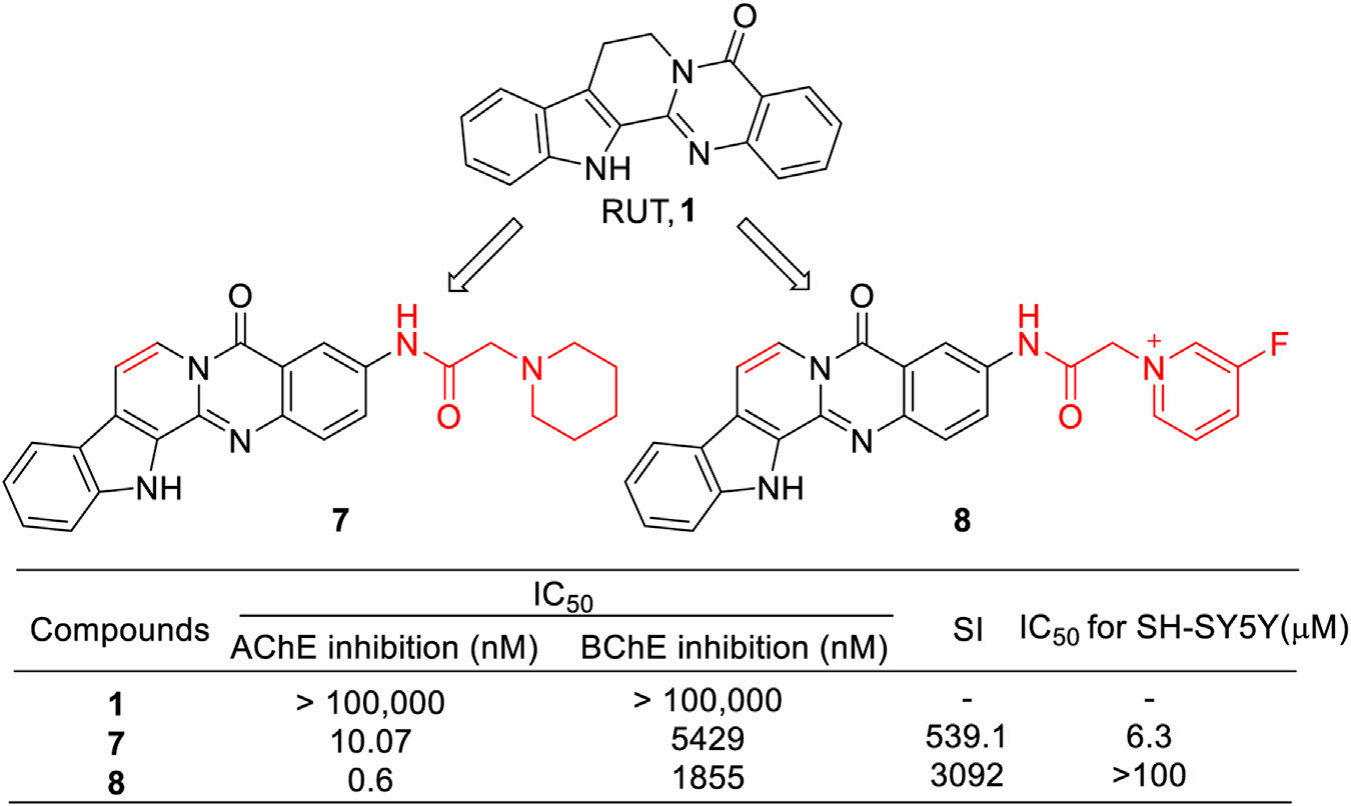
Structure and activities of the representative compounds **7** and **8**.

In 2010, the research group of Huang synthesized a series of novel RUT derivatives and evaluated their AChE inhibitory activity (Wang et al., 2010). Most of the derivatives were found to be selective for AChE over BChE. In particular, compound **7** showed the highest inhibitory activity toward AChE and also demonstrated the highest selectivity index (SI) over BChE. However, it showed a cytotoxic effect on SH-SY5Y cells. Considering that the dehydrorutaecarpine moiety had better potency than derivatives with the RUT moiety, they reported another series of dehydrorutaecarpine derivatives (He et al., 2013). All these synthetic compounds, particularly **8**, showed higher inhibitory activity against AChE, Aβ aggregation, and oxidative stress compared to RUT. Notably, **8** showed a lower cytotoxic effect on SH-SY5Y cells (Figure 7).

**FIGURE 8:**
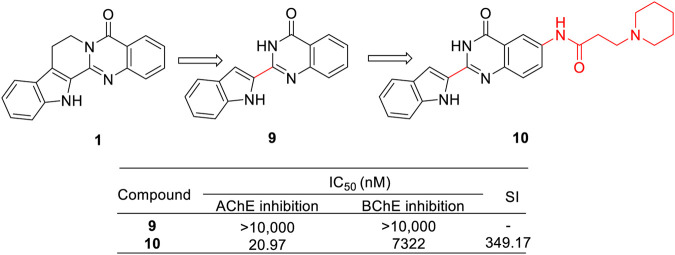
Structure and activities of the representative compounds **9** and **10**.

Another research group, Li et al. (2013), reported a series of novel 2-(2-indolyl-)-4 (3*H*)-quinazoline (2IQ, **9**) derivatives, which were regarded as the ring-opened analogs of RUT. Subsequently, they evaluated their pharmacological activity against AChE. Among them, **10** possessed the strongest inhibitory activity toward AChE with high selectivity over BChE (Figure 8).

#### 2.2.2 As PDE5 inhibitors

Phosphodiesterases (PDEs), being responsible for hydrolyzing two second messengers, cyclic GMP (cGMP) and cyclic AMP (cAMP), can affect neuronal cell survival (Tsunada et al., 2021). Thus, PDEs play important roles in neurodegenerative diseases, including AD (Zuccarello et al., 2020). Among the different PDEs, PDE5 specifically hydrolyzes cGMP. Recently, an upregulated PDE5 expression has been found in the brains of mild AD patients, and studies have confirmed that PDE5 inhibitors exhibit therapeutic effects on AD patients by improving learning and memory, and reversing cognitive impairment (Boccia et al., 2011; Hosseini-Sharifabad et al., 2012; Al-Amin et al., 2014). Therefore, several researchers are developing anti-AD drugs by reducing PDE5 levels.

**FIGURE 9:**
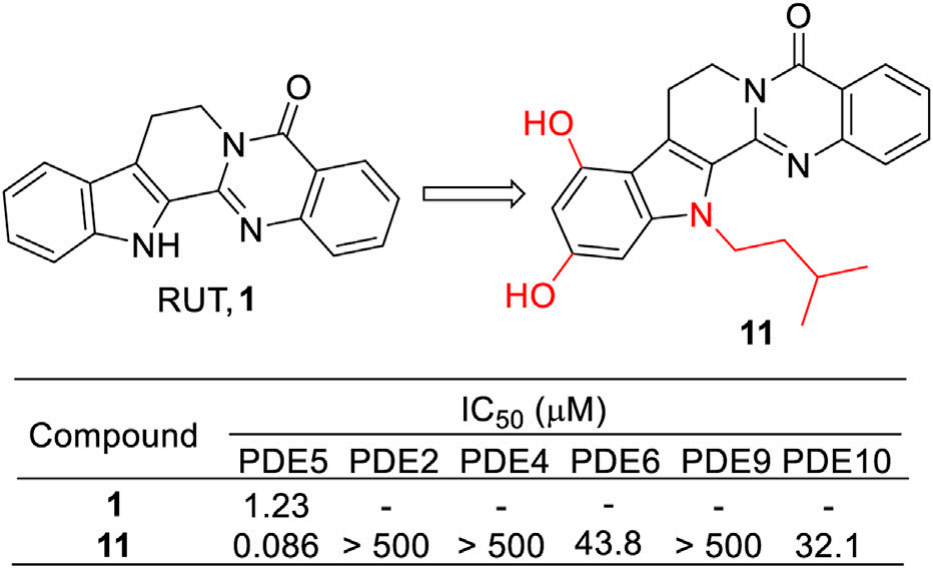
Structure and activities of the representative compound **11**.

Considering RUT containing a quinoline structure, which has been demonstrated to be a privileged structure for the PDE5 inhibitor (Bi et al., 2004), Huang et al. decided to use RUT as the lead compound to develop PDE5 inhibitors for the treatment of AD (Huang et al., 2020). Thus, a series of RUT derivatives were synthesized, and their PDE5 inhibitory activities were tested. As a result, **11** showed the most potent PDE5 inhibitory activity with an IC_50_ value of 0.086 μM. Moreover, it also exhibited good effects against scopolamine-induced cognitive impairment *in vivo*. These results might provide significant instruction for further developing PDE5 inhibitors derived from RUT (Figure 9).

### 2.3 Anti-tumor activity

#### 2.3.1 As topoisomerase inhibitors

**FIGURE 10:**
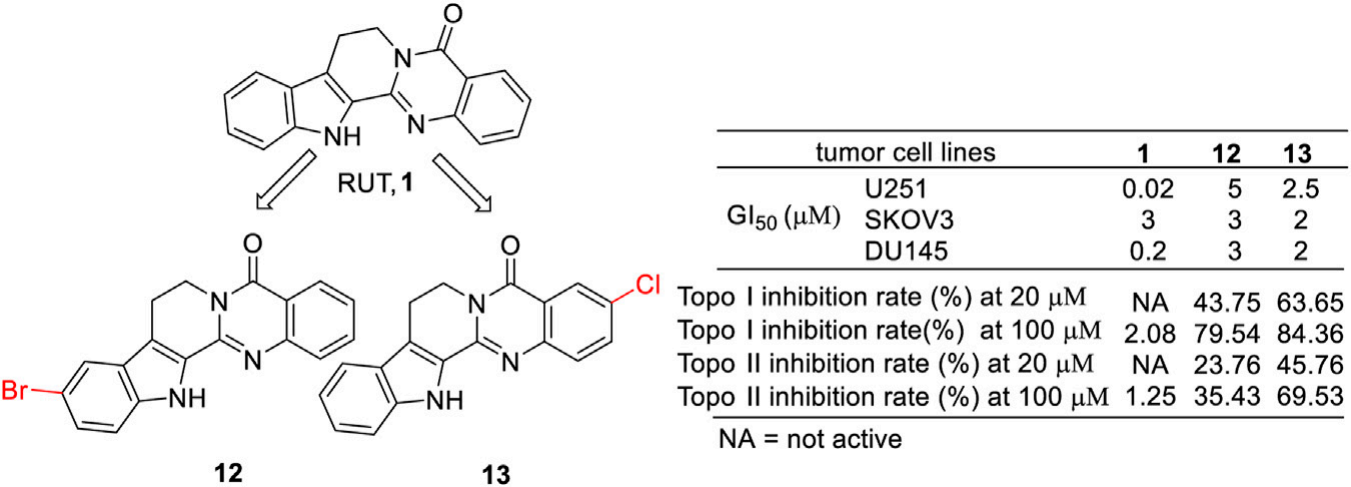
Structure and activities of the representative compounds **12** and **13**.

It is well known that topoisomerases are important targets in tumor therapy, and their inhibitors, including camptothecin, irinotecan, topotecan, etc., are widely used in clinic applications. In 2006, Xu et al. revealed that RUT itself did not show any significant activity against topoisomerases I and II (Topo I and Topo II) despite its significant cytotoxicity on several cancer cell lines (Xu et al., 2006). However, Kim et al. found that two derivatives of RUT (**12** and **13**) not only exhibited cytotoxicity against selected human cancer cell lines (GI_50_ values of 5 μM, 3 μM, and 3 μM for 15b against U251, SKOV3, and DU145 cell lines, respectively; GI_50_ values of 2.5 μM, 2 μM, and 2 μM for 15c against U251, SKOV3, and DU145 cell lines, respectively) (Baruah et al., 2004) but also showed inhibitory activities against Topo I and Topo II (Kim et al., 2012) (Figure 10).

#### 2.3.2 Targets undefined

**FIGURE 11:**
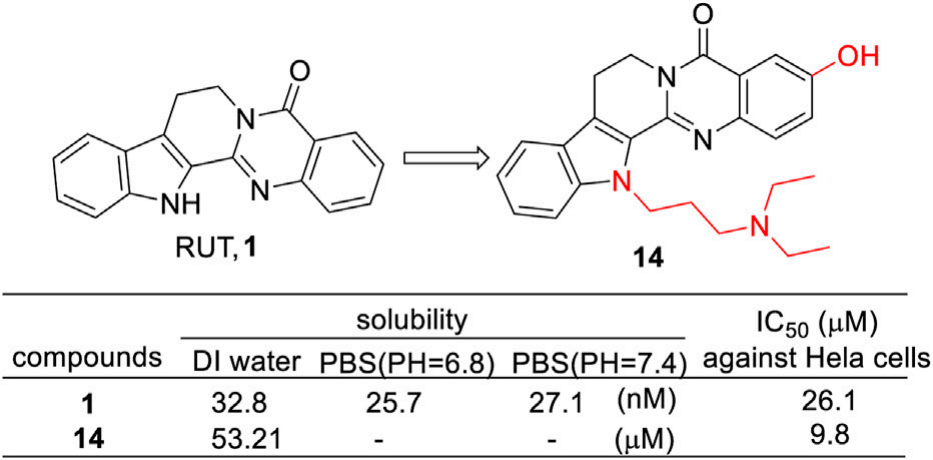
Structure and activities of the representative compound **14**.

Huang et al. synthesized two sets of RUT derivatives with amine groups to improve their solubility (Huang et al., 2017). The results showed that the solubility of the compounds with the amine moieties connected to the indole-*N* atom (**14**) was improved significantly. Moreover, cell viability on HeLa cells and reduction of metabolic activity were in the same range or better for these derivatives compared to the chemically unaltered parent compounds (Figure 11).

### 2.4 Antifungal activity

**FIGURE 12:**
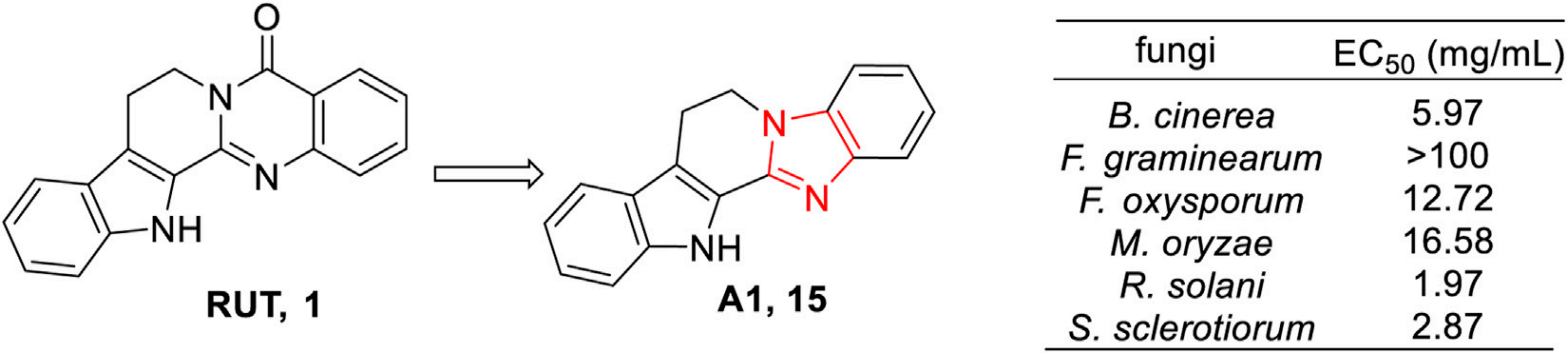
Structure and antifungal activity of the representative compound **15**.

To explore the potential applications of RUT and its derivatives against phytopathogenic fungi (*Botrytis cinerea*, *Fusarium graminearum*, *Fusarium oxysporum*, *Magnaporthe oryzae, Rhizoctonia solani*, and *Sclerotinia sclerotiorum*), various kinds of RUT derivatives were designed and synthesized by Yang et al. (2022), and their antifungal abilities against the aforementioned six phytopathogenic fungi were evaluated. The results showed that imidazole derivatives of RUT (**A1**, **15**) exhibited broad-spectrum inhibitory activities against *B. cinerea*, *F. graminearum*, *F. oxysporum*, *M. oryzae, R. solani*, and *S. sclerotiorum*, with EC_50_ values of 5.97 mg/mL, 12.72 mg/mL, 16.58 mg/mL, 1.97 mg/mL, and 2.87 mg/mL, respectively. However, RUT showed no activity against these six phytopathogenic fungi (the inhibition rate was 0% at 100 mg/mL). Moreover, the curative effects of **15** against *S*. *sclerotiorum* were 94.7%, 81.5%, 80.8%, and 65.0% at different concentrations of 400 mg/mL, 200 mg/mL, 100 mg/mL, and 50 mg/mL, respectively, in *in vivo* experiments, which was far more effective than the positive drug azoxystrobin. Notably, no phytotoxicity of **15** on oilseed rape leaves was observed even at a high concentration of 400 mg/mL. Therefore, **15** was considered a novel leading compound for the development of antifungal agents (Figure 12).

## 3 Conclusion

**FIGURE 13:**
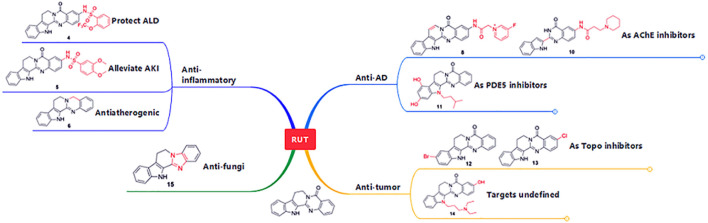
Summary of pharmacological activities and their targets possessed by RUT derivatives.

In summary, the diverse biological activities of RUT derivatives have been presented in this review. Undoubtedly, RUT and its derivatives exhibit diverse pharmacological activities, indicating that this class of alkaloids shows great potential in medicinal chemistry (Figure 13). Although there are currently no RUT-based drugs available in the market, research and development of RUT and its derivatives should be continued.

Nevertheless, the future directions of RUT and its derivatives could be summarized as follows: i) more studies should be carried out to clarify the similarities and differences between RUT and EVO. Both of them contain a pentacyclic scaffold, and their derivatives possess diverse pharmacological activities, including antitumor, anti-AD, and antifungal activities. However, based on the studies, it seems that RUT derivatives show better activity against inflammation and cardiovascular disease, while EVO derivatives show better activity against cancer. Zhang and coworkers explored the activity of the main bioactive indoloquinazoline alkaloids isolated from *Euodia rutaecarpa* on the aryl hydrocarbon receptor (AHR) (Zhang et al., 2018). The results showed that RUT was more efficient than EVO. These findings revealed that the methyl substitute at the *N*-14 atom was a key factor affecting AHR activation. However, similar studies addressing the underlying reasons for their different biological activities are rare. ii) The underlying reasons for one compound exhibiting different biological activities should be reinforced, as observed in the case of compounds **3** and **4**. In other words, detailed mechanism research related to the activity should be reinforced. iii) The structural modification of RUT to obtain a variety of sufficient derivatives still needs to be carried out: on one hand, to improve its physicochemical properties or selectivity, and on the other hand, to broaden the scope of compounds with diverse pharmacological activities. Moreover, SARs between different biological activities need to be summarized. iv) How to increase the bioavailability for clinical practice should be given more attention. Most of the compounds introduced in this article have not reached clinical trials; so, their physiological functions have not been clinically confirmed, which could mislead or cause bad effects.

## Abbreviations

AChE, acetylcholine; AD, Alzheimer’s disease; AKI, acute kidney injury; ALI, alcoholic liver disease; Aβ, amyloid β-protein; BChE, butyrylcholinesterase; CETSA, cellular thermal shift assay; COX-2, cyclooxygenase-2; EVO, evodamine; GI_50_, 50% growth inhibition concentration; IC_50_, 50% inhibition concentration; LPS, lipopolysaccharide; NLRP3, NOD-, LRR-, and pyrin domain-containing protein 3; NO, nitric oxide; PDE, phosphodiesterase; RUT, rutaecarpine; SAR, structure–activity relationship; SI, selectivity index; TNF-α, tumor necrosis factor-α; Topo, topoisomerase.
